# Supplementary material for: Rare transmission of commensal and pathogenic bacteria in the gut microbiome of hospitalized adults
Source: Nat Commun. 2022 Jan 31;13:586. doi: 10.1038/s41467-022-28048-7 (PMC8803835; doi:10.1038/s41467-022-28048-7)
Supplement: Supplementary file 1 — Supplementary information [file 41467_2022_28048_MOESM1_ESM.pdf]

Supplementary Information

Rare transmission of commensal and pathogenic bacteria in the gut microbiome of hospitalized adults

Siranosian et. al

Table of contents

Supplementary figures 3
Supplementary methods 12
Supplementary tables 15
Supplementary references 17

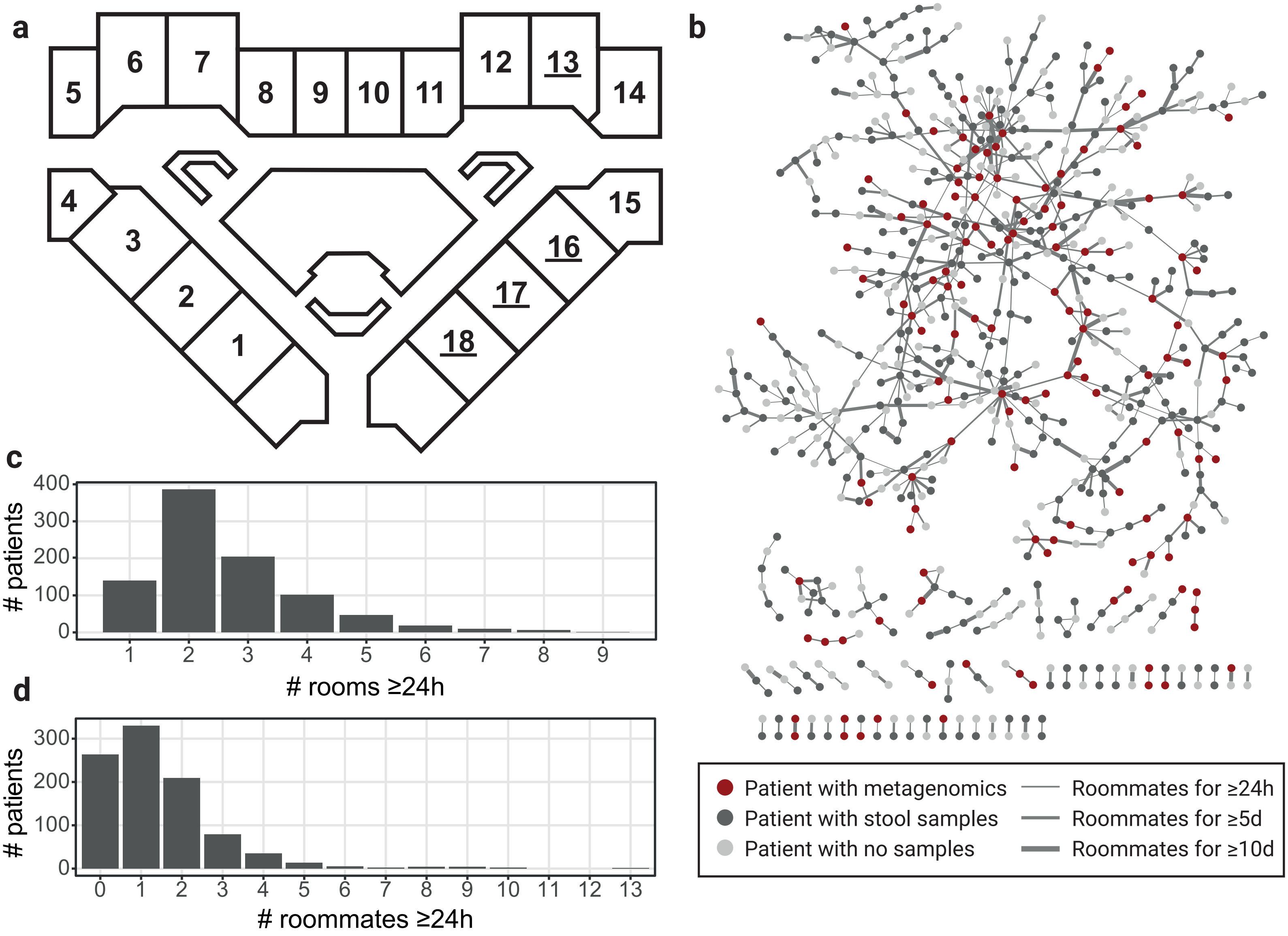

Figure S1: Analysis of hospital geography.
a) Layout of rooms in the HCT ward. Room numbers are indicated and double occupancy rooms are underlined.
b) Network view of patients who were roommates for at least 24 hours. Each node represents a single patient, colored according to if they have a banked stool sample or metagenomic sequencing data present. Edges are drawn between patients who were roommates, and edge width represents the length of overlap in the same room.
c) Histogram of the number of rooms patients occupied for at least 24 hours.
d) Histogram of the number of unique roommates patients had for at least 24 hours.

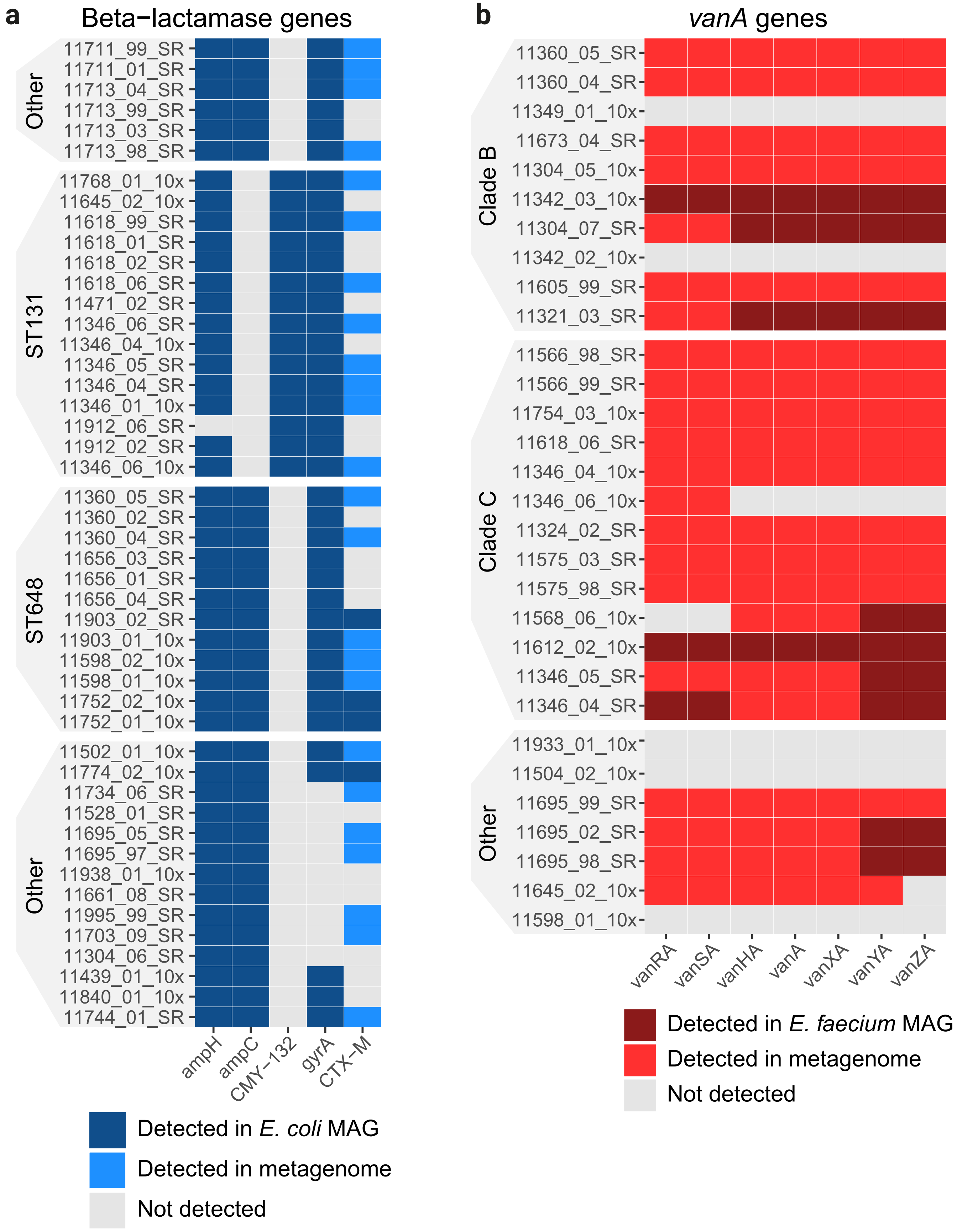

Figure S2: Antibiotic resistance genes detected. In each panel, samples are rows and resistance genes are columns. Samples are ordered and clades are highlighted corresponding to the respective figure in the main text. Cells are colored whether the gene was detected in the respective MAG from the sample, or just in the metagenome (indicating it may be on a plasmid).

a) Beta-lactamase genes detected in *E. coli* samples from Figure 2. The *gyrA* gene was detected with the CARD protein variant model, which requires a genetic variant conveying resistance in addition to the presence of the gene.

b) Vancomycin resistance genes of the *vanA* operon detected in *E. faecium* in samples from Figure 3.

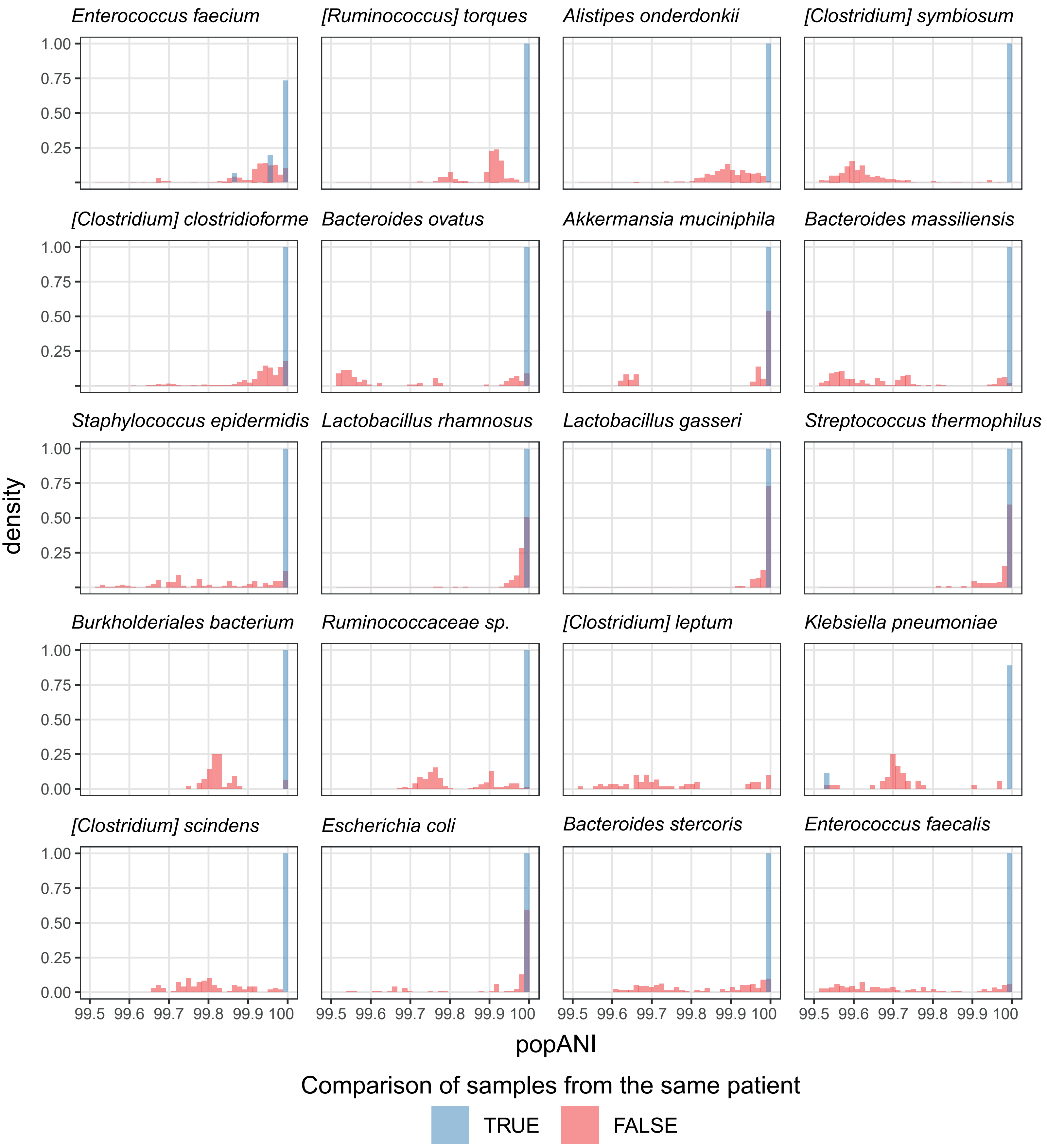

Figure S3: Distribution of popANI values comparing samples from the same or different patients. Distributions are split by species and the most common 25 species are shown. While in many cases the two distributions overlap, very rarely did popANI values comparing samples from different patients exceed the 99.999% transmission threshold. Comparisons with <99.5% popANI are omitted from the figure for clarity.

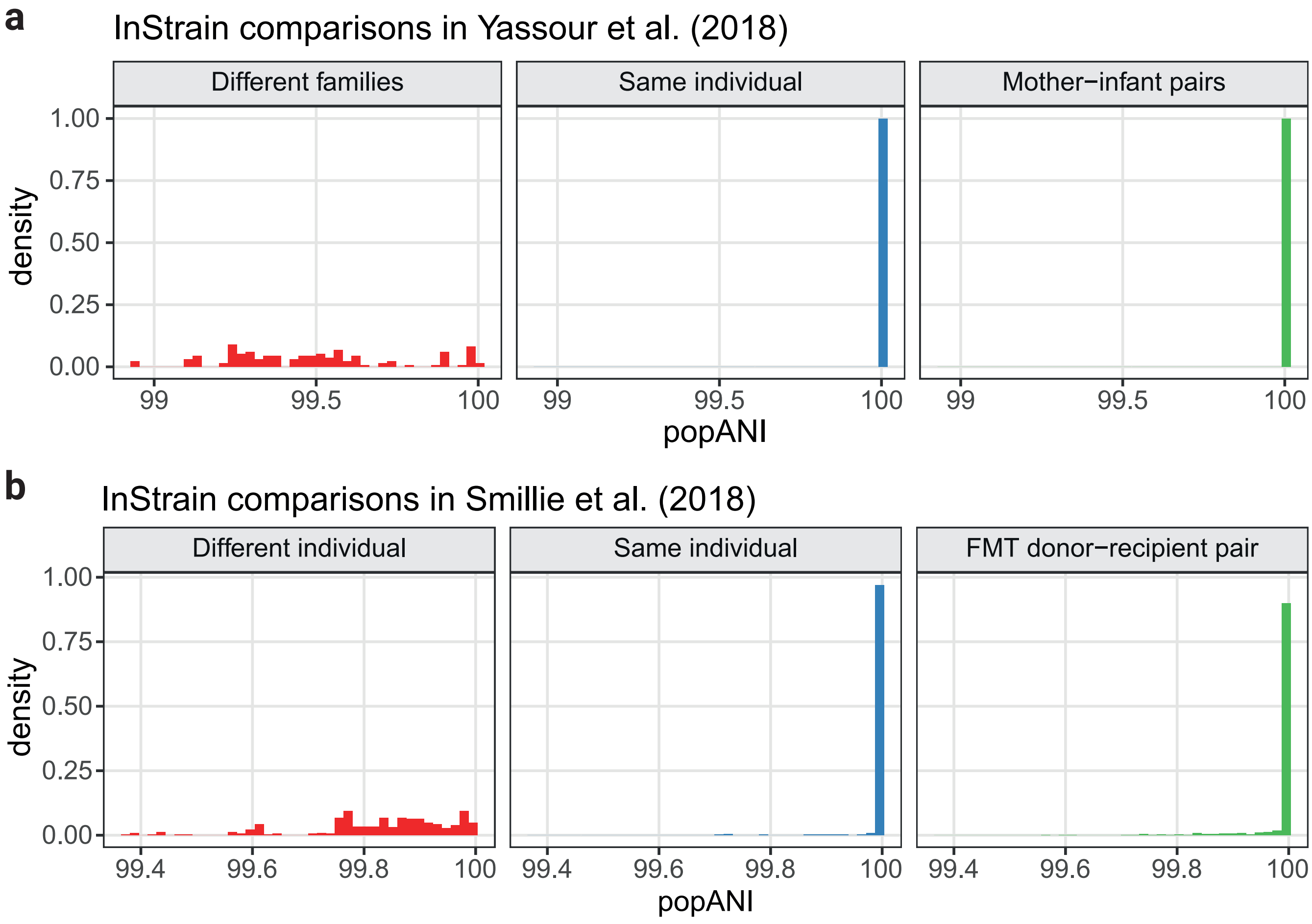

Figure S4: InStrain analysis of the five most common species in external datasets where transmission is expected to occur. Distributions of popANI values are separated based on the individuals the samples came from, with putative transmission events contained in the far right panel.
a) Metagenomic sequencing datasets from mother-infant pairs[18]. The maximum popANI value obtained when comparing samples from different families was 99.995%.
b) Metagenomic sequencing datasets from fecal microbiota transplantation donors and recipients[67]. The maximum popANI value obtained comparing samples from individuals not related by FMT was 99.998%.

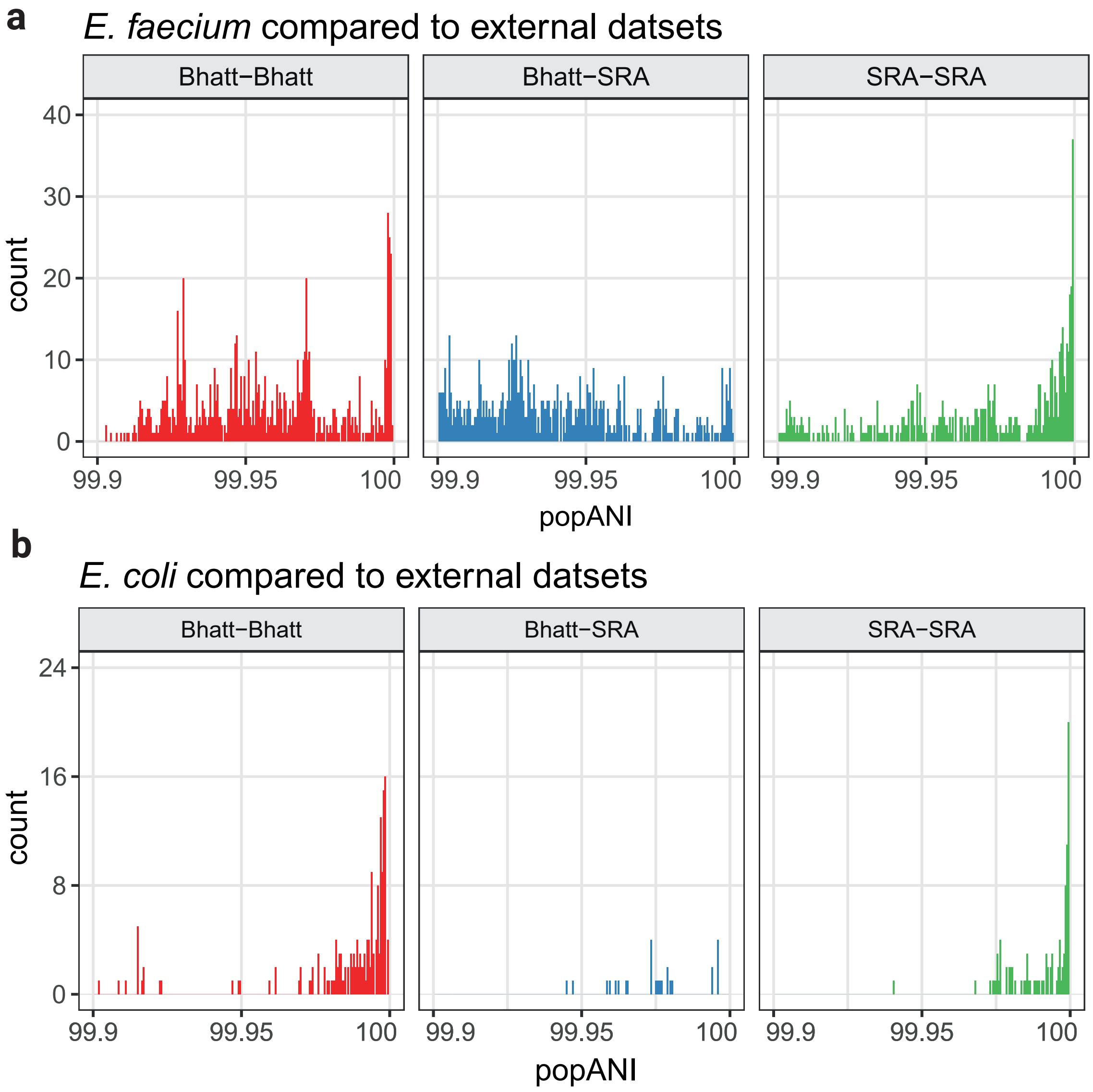

Figure S5: *Enterococcus faecium* (a) and *Escherichia coli* (b) strains compared to external datasets, including hospitalized adult and pediatric HCT patients, hospitalized infants and vancomycin-resistant *E. faecium* isolates[3,69-73]. Panels are separated according to whether comparisons were made within the data in this manuscript (Bhatt-Bhatt), between our data and external data (Bhatt-SRA) or within external data (SRA-SRA).

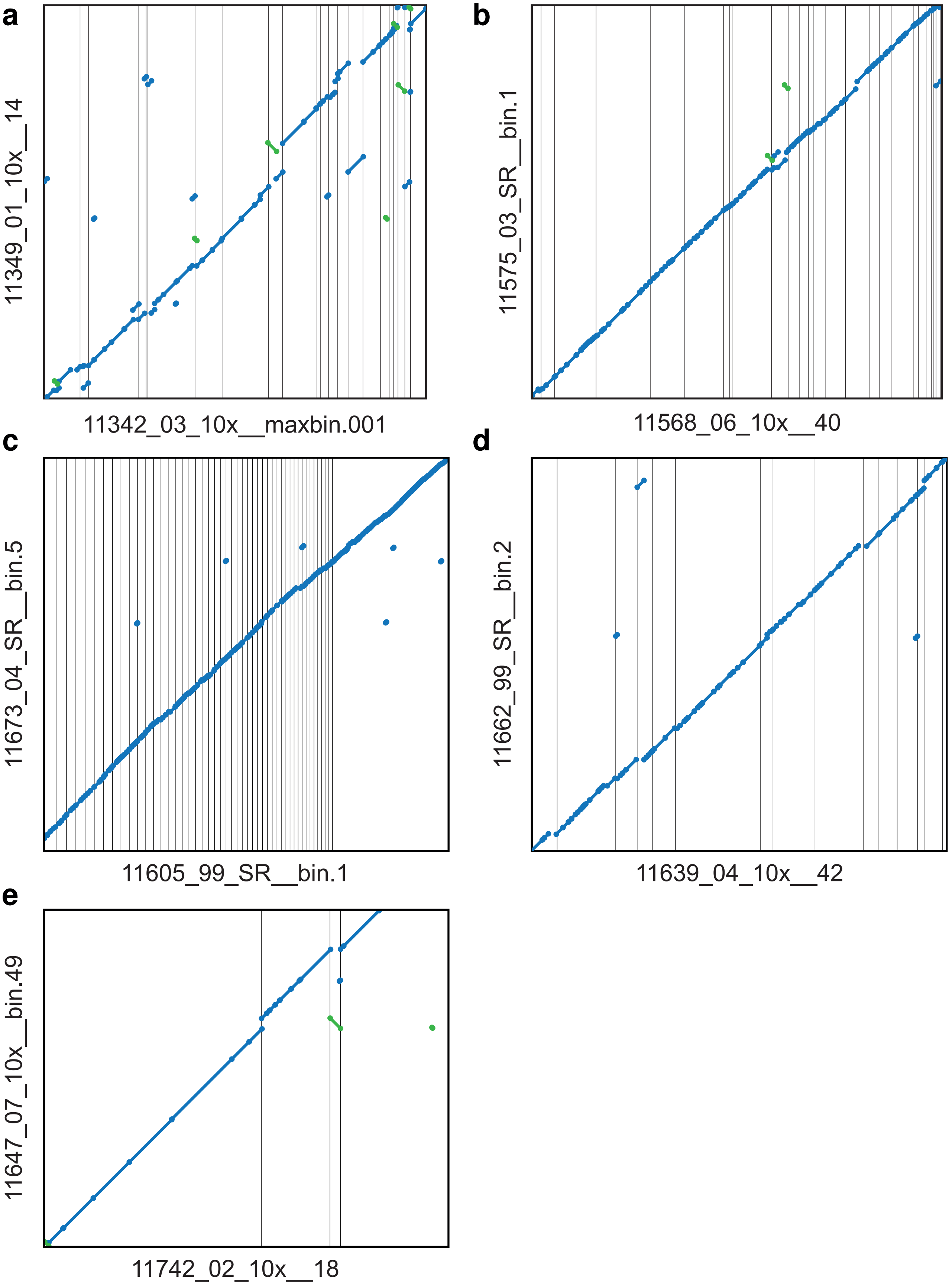

Figure S6: Dotplots showing pairwise alignment of MAGs in cases of putative transmission of the given species. Blue lines along the diagonal indicate 1-1 homology between the two sequences. Green lines indicate inversions that are likely the result of assembly or binning errors.

a) *E. faecium* MAGs from patients 11342 and 11349, corresponding to figure 4a.
b) *E. faecium* MAGs from patients 11575 and 11568, corresponding to figure 4b.
c) *E. faecium* MAGs from patients 11605 and 11673, corresponding to figure 4c.
d) *H. hathewayi* MAGs from patients 11639 and 11662, corresponding to figure 5a.
e) *A. muciniphila* MAGs from patients 11742 and 11647 corresponding to figure 5b.

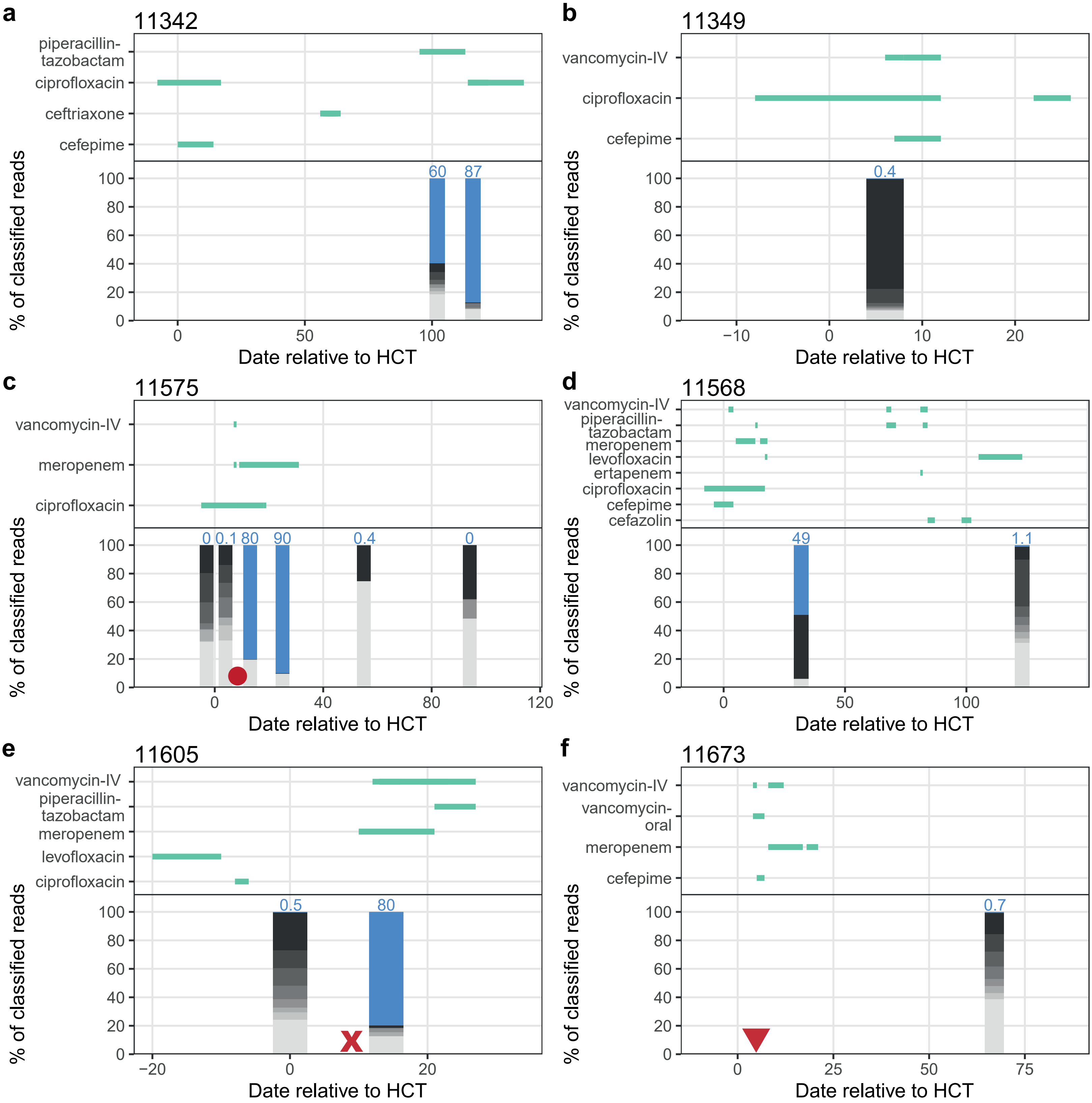

Figure S7: Antibiotic prescription and taxonomic composition of patients with nearly identical *Enterococcus faecium* strains. *E. faecium* abundance is shown in blue and indicated with text. Other taxa are shown in grey. All dates are relative to HCT for the particular patient. Approximate dates of BSI are shown with red symbols. Circle: *Klebsiella pneumoniae*, X: *Enterococcus faecium*, triangle: *Escherichia coli*.

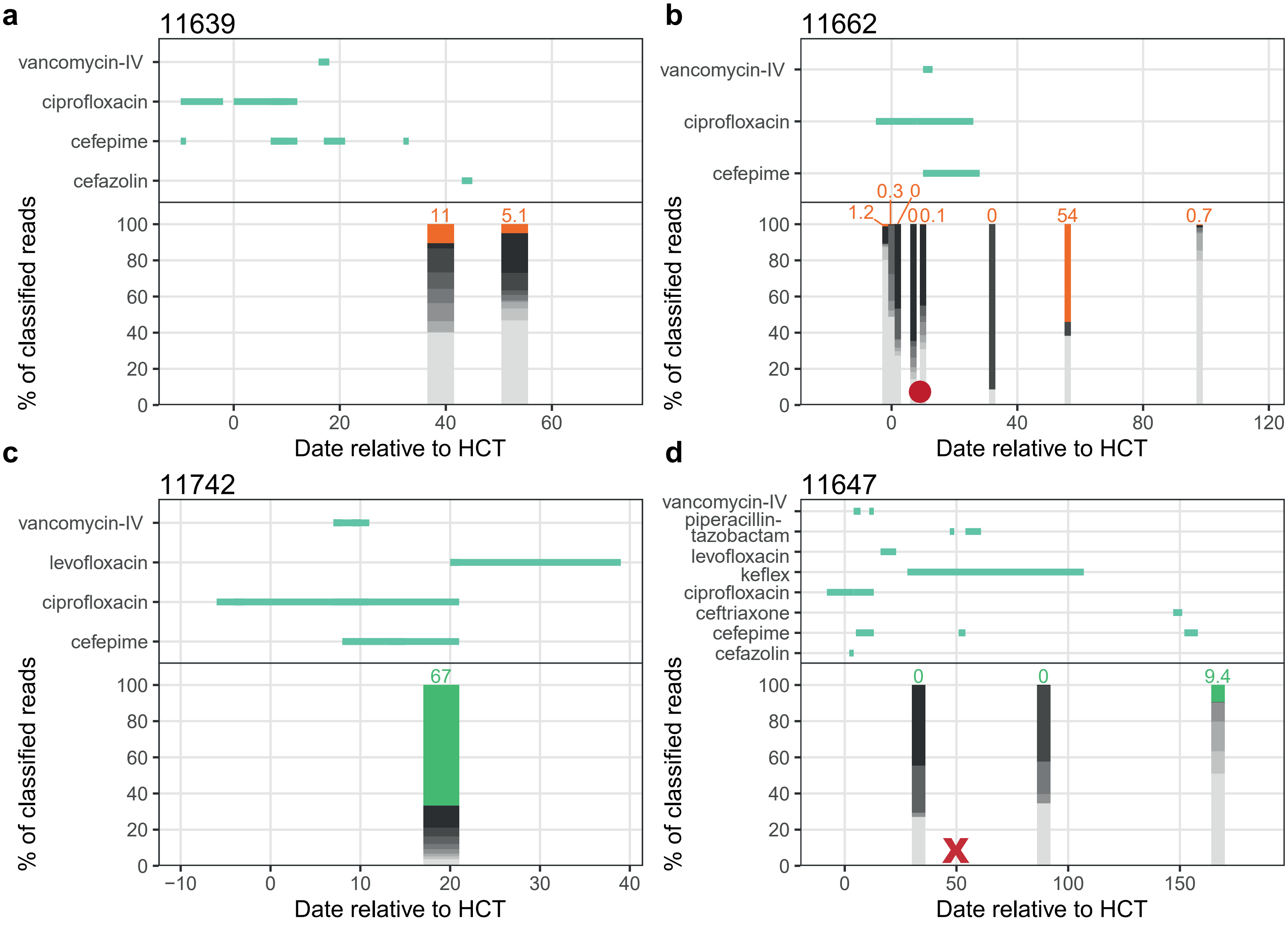

Figure S8: Antibiotic prescription and taxonomic composition of patients with nearly identical *Hungatella hathewayi* (shown in orange) or *Akkermansia muciniphila* (shown in green) strains. Other taxa are shown in grey. All dates are relative to HCT for the particular patient. Approximate dates of BSI are shown with red symbols. Circle: *Streptococcus mitis*, X: *Klebsiella pneumoniae.*

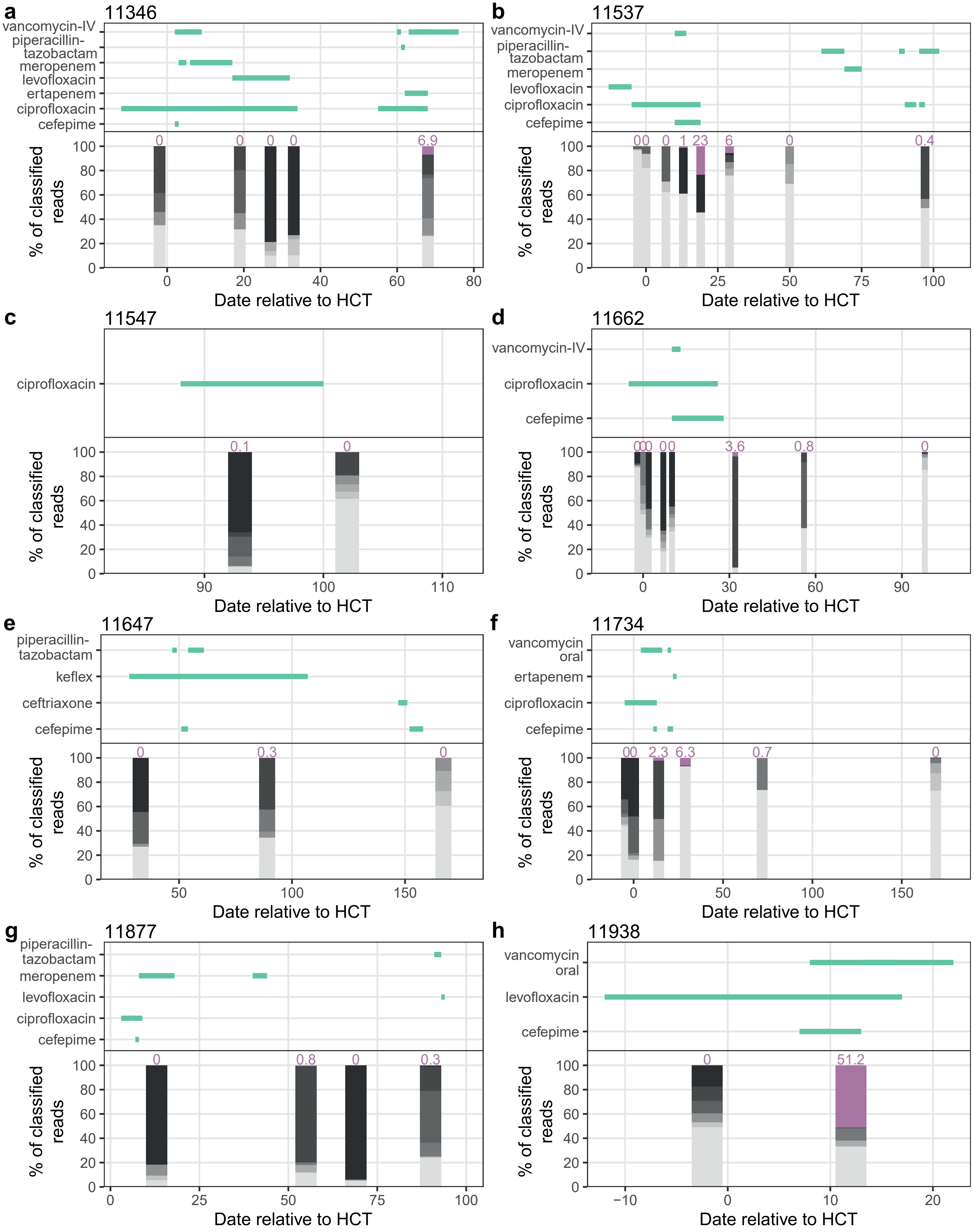

Figure S9: Antibiotic prescription and taxonomic composition of patients with nearly identical *Lactobacillus rhamnosus* strains. *L. rhamnosus* abundance is shown in purple and indicated with text. Other taxa are shown in grey. All dates are relative to HCT for the particular patient.

Supplementary methods: Mitigation of laboratory contamination and barcode swapping

Any study of transmission is susceptible to confounders that may introduce false positives. Two major sources are laboratory contamination and barcode swapping, both of which can make it appear as if identical strains are present in multiple samples. To minimize the chance of laboratory contamination, samples selected for linked-read sequencing were randomized prior to extraction into groups of 16, subject to the constraint that the number of samples from roommate pairs in the same extraction batch were minimized. These groupings were carried out through library preparation. Similar constraints were used when preparing pooled libraries for sequencing.

It is a recognized phenomenon that pooled Illumina sequencing libraries experience “barcode swapping” or “index hopping”[1] when libraries are differentiated by a single sample index. While this issue is avoided by using unique dual index sequences for all samples in a pool, our laboratory was not aware of the issue until 2018, and older libraries were prepared without a unique dual indexing strategy. Linked-read libraries only contain a single sample index sequence, which makes it impossible to eliminate the effect of barcode swapping, other than the costly option of devoting an entire lane to each sample.

In linked-read sequencing libraries, we were able to estimate the impact of barcode swapping. There are ~10 million possible 10X barcodes (these are the barcodes which convey long-range information, different from the sample index barcodes). While a subset of 10X barcodes will overlap between two samples, the fraction of barcodes from reads mapping to a single organism should be limited. We mapped reads from all linked-read samples against the uniquely identifiable p-crAssphage genome[2]. Then we looked at the fraction of 10X barcodes that overlapped between samples. Samples sequenced on different lanes typically had 0-30% 10X barcode overlap. Samples sequenced on the same lane had 60-100% of barcode overlap

in some cases. We set a threshold of 40% overlap of barcode sets to call a comparison "swapped" and remove it from analysis. By counting reads believed to be assigned to improper samples because of barcode swapping, we estimate the rate in our linked-read data to be 0.1-0.2%.

While this rate may seem small, at high sequencing depth and with abundant organisms, it quickly results in enough reads being swapped to assemble a genome or conduct an inStrain comparison. Indeed, we found cases where multiple species (instead of the single species believed to be the result of transmission events) were shared between linked-read samples sequenced on the same lane that were likely the result of barcode swapping.

We also attempted to measure the degree of barcode swapping in dual-indexed lanes of short read Illumina sequencing. Using the uniquely identifiable p-crAssphage genome as a marker for swapping, we observed roughly 0.5% of sequencing reads swapped between samples on the same lane that shared one index sequence. Samples on the lane that shared no sequencing indices often had p-crAssphage below 1e-5%. Simple relative abundance metrics cannot distinguish between barcode swapping and a true difference in abundance between samples. However, even with the 0.5% rate of swapping, we regularly observed >5x coverage of the p-crAssphage genome in what we believe to be the truly negative samples, and the resulting inStrain comparisons revealed sufficient paired genome coverage and 100% popANI. We never observed identical p-crAssphage genomes between samples from different patients sequenced with unique dual indices or on different lanes.

For short-read sequencing samples, we know which pairs of samples share one of two index sequences and have the possibility of being impacted by swapping. We cannot estimate the impact of barcode swapping like was done for linked-read datasets. We simply eliminated all comparisons where two samples had the possibility of barcode swapping, and all comparisons that could be affected by "secondary" swapping, where the samples were not directly affected, but an interaction between other samples from the two patients could cause false positives.

While this filtering may discard legitimate transmission events, we believe it is necessary to lower the number of false positives.

Previous DNA extraction and short-read sequencing efforts did not follow the randomization constraints above and we cannot guarantee that laboratory contamination did not happen at some point in the process. However, we note that cases of laboratory contamination or barcode swapping would result in the entire microbiome composition of one sample being transferred to another. After our stringent filters, we only discovered one case where patients shared two separate species. As these were both *Lactobacillus* species, our hypothesis about probiotic consumption is a possible explanation.

Organism	HCT patients (2016-2018)	Hospital-wide (2018)
Escherichia coli	18	196
Viridans group Strep	18	39
Enterococcus sp.	12	98
Staphylococcus aureus	4	132
Pseudomonas aeruginosa	3	9
Klebsiella pneumoniae	2	74
Enterobacter sp.	2	27
All other	6	271

Supplementary Table 1: Count of BSIs in HCT patients or hospital-wide, by organism.

name	species	NCBI source	reference
**536**	E. coli	https://www.ncbi.nlm.nih.gov/assembly/GCF_000013305.1/	
**APEC_O1**	E. coli	https://www.ncbi.nlm.nih.gov/assembly/GCF_003028815.1/	
**BIDMC_43b**	E. coli	https://www.ncbi.nlm.nih.gov/assembly/GCF_000026325.2/	
**CFT073**	E. coli	https://www.ncbi.nlm.nih.gov/assembly/GCF_003028795.1/	
**E2348_69**	E. coli	https://www.ncbi.nlm.nih.gov/assembly/GCF_000026545.1/	
**EC958**	E. coli	https://www.ncbi.nlm.nih.gov/assembly/GCF_000285655.3/	Forde et al. 2014
**ED1a**	E. coli	https://www.ncbi.nlm.nih.gov/assembly/GCF_000026305.1/	
**IMT16316**	E. coli	https://www.ncbi.nlm.nih.gov/assembly/GCF_002587005.1/	Schaufler et al. 2019
**JJ1886**	E. coli	https://www.ncbi.nlm.nih.gov/assembly/GCF_000493755.1/	Andersen et al. 2016
**SE15**	E. coli	https://www.ncbi.nlm.nih.gov/assembly/GCF_000010485.1/	
**UTI89**	E. coli	https://www.ncbi.nlm.nih.gov/assembly/GCF_000013265.1/	
**E1**	E. faecium	https://www.ncbi.nlm.nih.gov/assembly/GCF_001886635.1/	Tedim et al. 2017
**EF_508**	E. faecium	https://www.ncbi.nlm.nih.gov/assembly/GCF_004152205.1/	
**EF_510**	E. faecium	https://www.ncbi.nlm.nih.gov/assembly/GCF_004152185.1/	
**EF_517**	E. faecium	https://www.ncbi.nlm.nih.gov/assembly/GCF_004152105.1/	
**IHC5**	E. faecium	https://www.ncbi.nlm.nih.gov/assembly/GCF_002158325.1/	

Supplementary Table 2: Reference genomes analyzed, corresponding to Figure 3 and Figure 4a.

Supplementary references

[1] Illumina, Inc. Minimizing Index Hopping. https://www.illumina.com/techniques/sequencing/ngs-library-prep/multiplexing/index-hopping.html (2018).

[2] Dutilh, B. E. et al. A highly abundant bacteriophage discovered in the unknown sequences of human faecal metagenomes. Nat. Commun. 5, 4498 (2014).
