# Supplementary material for: Rare transmission of commensal and pathogenic bacteria in the gut microbiome of hospitalized adults
Source: Nat Commun. 2022 Jan 31;13:586. doi: 10.1038/s41467-022-28048-7 (PMC8803835; doi:10.1038/s41467-022-28048-7)
Supplement: Supplementary file 3 — Description of Supplementary Data Files [file 41467_2022_28048_MOESM3_ESM.docx]

Description of supplementary data files

Rare transmission of commensal and pathogenic bacteria in the gut microbiome of hospitalized adults

Siranosian et al.

**Supplementary Data 1:** List and ages of patients with samples investigated in this study.

**Supplementary Data 2:** Sequencing datasets analyzed in this study.

**Supplementary Data 3:** Statistics on MAGs generated in this study.

**Supplementary Data 4:** Kraken2 classification results for all samples at the species level. Species with less than 0.01% abundance have been removed.

**Supplementary Data 5:** Kraken2 classification results for all samples at the genus level. Genera with less than 0.01% abundance have been removed.

**Supplementary Data 6:** Antibiotic prescription data for each patient, limited to ∓ 100 days relative to HCT.

**Supplementary Data 7:** Matrix of number of days patients overlapped in the hospital.

**Supplementary Data 8:** Matrix of number of days patients overlapped as roommates.

**Supplementary Data 9:** InStrain results for all comparisons made between samples in this manuscript. Filtered to remove comparisons <99.99% popANI, <0.5 percent_compared and potential barcode swapping results.

**Supplementary Data 10:** Pairwise alignment-based ANI for Escherichia coli and Enterococcus faecium genomes analyzed in Figures 2 and 3. MAGs are identified by a concatenation of the seq_id and Bin columns in Supplementary Data 3.
