# Supplementary material for: Rare transmission of commensal and pathogenic bacteria in the gut microbiome of hospitalized adults
Source: Nat Commun. 2022 Jan 31;13:586. doi: 10.1038/s41467-022-28048-7 (PMC8803835; doi:10.1038/s41467-022-28048-7)
Supplement: Supplementary file 14 — Reporting Summary [file 41467_2022_28048_MOESM14_ESM.pdf]

nature research

Corresponding author(s): Ami S. Bhatt

Last updated by author(s): Dec 12, 2021

Reporting Summary

Nature Research wishes to improve the reproducibility of the work that we publish. This form provides structure for consistency and transparency in reporting. For further information on Nature Research policies, see our Editorial Policies and the Editorial Policy Checklist.

Statistics

For all statistical analyses, confirm that the following items are present in the figure legend, table legend, main text, or Methods section.

n/a	Confirmed	
☐	☒	The exact sample size (n) for each experimental group/condition, given as a discrete number and unit of measurement
☒	☐	A statement on whether measurements were taken from distinct samples or whether the same sample was measured repeatedly
☐	☒	The statistical test(s) used AND whether they are one- or two-sided<br>*Only common tests should be described solely by name; describe more complex techniques in the Methods section.*
☐	☒	A description of all covariates tested
☒	☐	A description of any assumptions or corrections, such as tests of normality and adjustment for multiple comparisons
☐	☒	A full description of the statistical parameters including central tendency (e.g. means) or other basic estimates (e.g. regression coefficient) AND variation (e.g. standard deviation) or associated estimates of uncertainty (e.g. confidence intervals)
☐	☒	For null hypothesis testing, the test statistic (e.g. F, t, r) with confidence intervals, effect sizes, degrees of freedom and P value noted<br>*Give P values as exact values whenever suitable.*
☒	☐	For Bayesian analysis, information on the choice of priors and Markov chain Monte Carlo settings
☒	☐	For hierarchical and complex designs, identification of the appropriate level for tests and full reporting of outcomes
☐	☒	Estimates of effect sizes (e.g. Cohen's d, Pearson's r), indicating how they were calculated

Our web collection on statistics for biologists contains articles on many of the points above.

Software and code

Policy information about availability of computer code

Data collection	No software was used to collect data in this study.
Data analysis	We used the following software for data analysis. details are provided in the study methods.<br>TrimGalore version 0.5.0, SeqKit version 0.9.1, BWA version 0.7.17-r1188, FastQC version 0.11.8, Kraken2 version 2.0.8-beta, Bracken version 2.0, SPAdes version 3.14.0, Megahit version 1.2.9, Athena version 1.3, Metabat2 version 2.15, Maxbin version 2.2.7, CONCOCT version 1.1.0, DASTool version 1.1.1, CheckM version 1.0.13, dRep version 2.6.2, inStrain version 1.3.11, MUMmer version 4.0.0beta2, R version 4.0.3, Resistance Gene Identifier version 5.1.1, vegan version 2.5-7, dot version N/A.

For manuscripts utilizing custom algorithms or software that are central to the research but not yet described in published literature, software must be made available to editors and reviewers. We strongly encourage code deposition in a community repository (e.g. GitHub). See the Nature Research guidelines for submitting code & software for further information.

Data

Policy information about availability of data

All manuscripts must include a data availability statement. This statement should provide the following information, where applicable:

- Accession codes, unique identifiers, or web links for publicly available datasets
- A list of figures that have associated raw data
- A description of any restrictions on data availability

Raw sequence data for this manuscript, when not previously published, have been uploaded to NCBI SRA under project number PRJNA707487 [https://www.ncbi.nlm.nih.gov/bioproject/?term=PRJNA707487]. MAGs generated in this study are available from Zenodo in a tar.gz archive (8.4 Gb) under record number 5768708 [https://zenodo.org/record/5768708]. Information on reference genomes used when building trees have been provided in supplementary table 1. Additional information on patient clinical metadata, sequencing datasets generated, statistics of MAGs generated, kraken2 classification results, ANI and inStrain

results, days of overlap between patients and antibiotic prescription are available as supplementary data files. Additional potentially identifying patient clinical information is available from the corresponding author upon reasonable request. The kraken2 classification database was built from genomes contained in NCBI Genbank [https://www.ncbi.nlm.nih.gov/genbank/]. The Comprehensive Antibiotic Resistance Database is available online [https://card.mcmaster.ca/].

Field-specific reporting

Please select the one below that is the best fit for your research. If you are not sure, read the appropriate sections before making your selection.

☒ Life sciences ☐ Behavioural & social sciences ☐ Ecological, evolutionary & environmental sciences

For a reference copy of the document with all sections, see nature.com/documents/nr-reporting-summary-flat.pdf

Life sciences study design

All studies must disclose on these points even when the disclosure is negative.

Sample size	Samples were selected for analysis based on the availability of biobanked stool samples. No sample size calculation was conducted prior to the study.
Data exclusions	Samples where we failed to extract enough high-molecular weight DNA for sequencing were excluded. No data were excluded following sequencing.
Replication	Findings were not replicated for this study, but we retain all original biological material and can reproduce if necessary. No findings were attempted to be replicated unsuccessfully.
Randomization	Prior to DNA extraction and sequencing, samples were randomized to mitigate the effect of laboratory contamination (if any). In the only other experimental work, Isolation, culture and identification of VRE organisms, only three samples were analyzed and randomization was not possible.
Blinding	Blinding was not done for this study. We do not believe it was necessary, as there were no treatment conditions. It was also necessary for investigators to know patient identifiers to differentiate putative transmission events from time course samples from a single patient.

Reporting for specific materials, systems and methods

We require information from authors about some types of materials, experimental systems and methods used in many studies. Here, indicate whether each material, system or method listed is relevant to your study. If you are not sure if a list item applies to your research, read the appropriate section before selecting a response.

Materials & experimental systems

n/a	Involved in the study	
☒	☐	Antibodies
☒	☐	Eukaryotic cell lines
☒	☐	Palaeontology and archaeology
☒	☐	Animals and other organisms
☐	☒	Human research participants
☒	☐	Clinical data
☒	☐	Dual use research of concern

Methods

n/a	Involved in the study	
☒	☐	ChIP-seq
☒	☐	Flow cytometry
☒	☐	MRI-based neuroimaging

Human research participants

Policy information about studies involving human research participants

Population characteristics	The study population was adult patients undergoing hematopoietic cell transplantation (HCT) for the treatment of an underlying hematological condition. Detailed characteristics of the patient population are available in Table 1, and per-patient metadata is contained in supplementary data 1. Patients ranged from age 20-72. The cohort was 58% male.
Recruitment	All hospitalized HCT patients during the study period were asked to consent to sample collection. Patients who produced at least one stool sample were available for analysis. Samples were selected for sequencing based on patients who had roommates with samples spanning the overlap period. Patients with severe graft versus host disease or other complications may produce samples that do not have sufficient DNA following extraction, leading to these patients being possibly under-represented in the dataset.
Ethics oversight	The Stanford University Research Compliance Office approved the IRB protocol (#8903; Principal Investigator: Dr. David Miklos, co-Investigator: Drs. Ami Bhatt and Tessa Andermann)

Note that full information on the approval of the study protocol must also be provided in the manuscript.
